# Effects of 16-Form Wheelchair Tai Chi on the Autonomic Nervous System among Patients with Spinal Cord Injury

**DOI:** 10.1155/2020/6626603

**Published:** 2020-11-27

**Authors:** Yan Qi, Haixia Xie, Yunlin Shang, Lejun Wang, Ce Wang, Yalin He, Wenxin Niu, Dongliang Shi

**Affiliations:** ^1^Yangzhi Rehabilitation Hospital, Tongji University School of Medicine, Shanghai 201619, China; ^2^Department of Rehabilitation Therapy, Tongji University School of Medicine, Shanghai 200092, China; ^3^Department of Physical Education, Tongji University, Shanghai 200092, China

## Abstract

**Objective:**

This study aims to investigate the effects of 16-form Wheelchair Tai Chi (WCTC16) on the autonomic nervous system among patients with spinal cord injury (SCI).

**Methods:**

Twenty patients with chronic complete thoracic SCI were recruited. Equivital life monitoring system was used to record and analyze heart rate variability (HRV) of patients for five minutes before and after five consecutive sets of WCTC16, respectively. The analysis of HRV in the time domain included RR intervals, the standard deviation of all normal RR intervals (SDNN), and the root mean square of the differences between adjacent NN intervals (RMSSD). The analysis of HRV in the frequency domain included total power (TP), which could be divided into very-low-frequency area (VLFP), low-frequency area (LFP), and high-frequency area (HFP). The LF/HF ratio as well as the normalized units of LFP (LFPnu) and HFP (HFPnu) reflected the sympathovagal balance.

**Results:**

There was no significant difference in RR interval, SDNN, RMSSD, TP, HEP, VLFP, and LFP of SCI patients before and after WCTC16 exercise (*P* > 0.05). LFPnu and HF peak decreased, while HFPnu and LF/HF increased in SCI patients after WCTC16 exercise. The differences were statistically significant (*P* < 0.001).

**Conclusion:**

WCTC16 can enhance vagal activity and decrease sympathetic activity so that patients with chronic complete thoracic SCI can achieve the balanced sympathovagal tone.

## 1. Introduction

Spinal cord injury (SCI) refers to damage to the structure and function of the spinal cord due to various reasons, resulting in sensory, motor, and autonomic dysfunction below the injury level [1]. Autonomic dysfunction after SCI is a common complication with an incidence of 43% [2], which can lead to stroke, convulsions, cardiac arrest, and death [3].

Autonomic dysfunction is a potentially life-threatening complication that occurs in patients with SCI [4]. Autonomic disorders are currently the main cause of death in patients with chronic SCI [5]. Some scholars have pointed out that autonomic dysfunction can reach a relatively “internally stable” state through complex adjustments in patients with chronic SCI [6–8]. For example, after the sympathetic innervation of the heart in patients with chronic quadriplegia is interrupted, the brain can also send signals that inhibit the activity of the parasympathetic nerve in order to maintain the homeostasis of the sympathetic-vagus nerve and simultaneously provoke reduction of sympathetic and parasympathetic nerve activity meaning autonomic reflex disorders [7].

Exercise is particularly important for the patients with SCI for that it can promote the recovery of neurological function and functional compensation, improve motor function and activities of daily living, enhance cardiovascular and respiratory functions, and develop balance function [9–13]. However, inappropriate exercise can induce autonomic disorders. It has been reported that about 90% of tetraplegic athletes induce abnormal autonomic reflexes in order to increase performance [14].

Tai Chi is a treasure of Chinese culture and has been widely applied in rehabilitation [15, 16]. A large number of studies have confirmed that traditional Tai Chi exercises can improve the cardiopulmonary function of practitioners [17, 18], but the mechanism of autonomic nervous system regulation that improves cardiovascular function is still unclear, and the results were often controversial [19, 20].

Because traditional Tai Chi is performed in a standing posture, many practitioners have difficulty in completing it due to various reasons. Therefore, based on the essence of traditional Tai Chi, wheelchair Tai Chi was proposed. It retains the movements of the upper limbs and torso and reduces or even does not have the movements of the lower limbs. It is suitable for a wider range of people, especially for the patients with SCI [21]. Similarly, it is unclear whether wheelchair Tai Chi can enhance the balance of autonomic nerve activity in chronic SCI.

In terms of autonomic nerve function assessment, heart rate variability (HRV), the 24-hour sinus heart rate having a certain degree of change over time, is getting more and more attention. HRV reflects the activity of the autonomic nervous system and quantitatively evaluates the tension and balance of the cardiac sympathetic nerve and vagus nerve, thereby revealing the regulation or influence of the autonomic nervous system on the cardiovascular system. HRV is a reliable, sensitive, and repeatable noninvasive detection method in SCI [22, 23]. Its time domain and frequency domain analysis method are basically mature in theory, and its clinical application range is wide. The frequency domain method can quantitatively indicate the modulation of the heart by the sympathetic nerve and the vagus nerve.

This study investigated the HRV indicators to explore the effect of 16-form Wheelchair Tai Chi (WCTC16) on the autonomic nervous system of patients with SCI in order to provide theoretical basis for safe and effective scientific fitness.

## 2. Methods

### 2.1. Participant Recruitment

The immediate effect of WCTC16 on autonomic nervous modulation in patients with chronic SCI was investigated. Twenty patients with complete thoracic SCI were recruited. These participants were right-handed SCI inpatients recruited during their recovery period from a rehabilitation hospital. The inclusion criteria were people who met the diagnostic criteria for complete SCI according to the American Spinal Injury Association [24]. They were all between 20 and 60 years old, able to communicate and follow instructions, and able to maintain a sitting posture for more than 30 minutes in a wheelchair.

The exclusion criteria were people with an unstable spine, metastatic cancer, spine tumor, serious cardiopulmonary disease, poorly controlled hypertension, poorly controlled trunk and upper limb hypertonia, or serious complications related to SCI, such as pressure ulcers and contracture.

The protocol was approved by the ethics committee of the rehabilitation, hospital and written informed consent was obtained from all participants before study. This study was registered on the Chinese Clinical Trial Registry (ChiCTR-1900023734).

### 2.2. Test Equipment

The Equivital series of dynamic vital signs monitors (Equivital Life Monitor HIDA3330-13-1P16, UK) monitored the participants' real-time heart rate, respiratory rate, body position, and body temperature; analyzed the HRV RR interval and the standard deviation of normal RR interval (SDNN), total power (TP), low-frequency power (LFP), high-frequency power (HFP), standardized HF, and other indicators of the changes using the built-in software; and then inferred the regulatory mechanism of the autonomic nervous system (sympathetic nerve and vagus nerve). The reliability and validity of the instrument had been confirmed [23].

### 2.3. Testing Method

Participants should avoid strenuous exercise within 24 hours before the test and smoking or drinking during the 2 hours before the test. They should have proper rest about 30 minutes and no full meals. Loose clothing was suitable for exercise during each test. After the participants arrived at the test site, they sat quietly to restore their physical functions. When they were sitting quietly, they were instructed to breathe calmly and steadily, stay in the surrounding environment away from noise interference, forbid talking with the participants, and keep the posture as relaxed and stable as possible.

**Figure 1:**
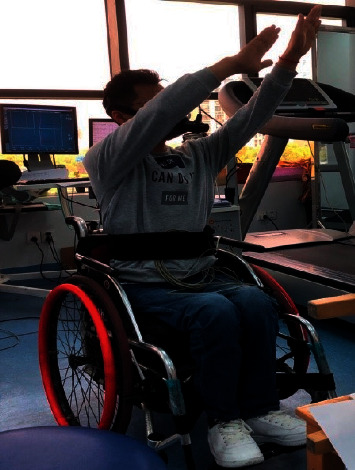
Dynamic vital sign monitor during 16-form Wheelchair Tai Chi.

Then, they wore a dynamic vital sign monitor, and HRV indicators were collected in a resting state for 5 minutes, as shown in Figure 1. The participants completed the warm-up with music, five consecutive WCTC16 exercises, and finishing exercises. After the recovery period, HRV indicators were collected for 5 minutes. After the test, all the data were imported into the automatic analysis software to explore the regulation mechanism of autonomic nerve function during the resting or pre-WCTC16 period before exercise and during the recovery or post-WCTC16 period after exercise. The rhythm and duration of WCTC16 exercises were controlled by 5-minute Tai Chi music with action prompts.

### 2.4. Test Parameters

#### 2.4.1. Time Domain Index of HRV

  RR interval: the average value of the normal RR interval, in ms
  SDNN: the standard deviation of the normal RR interval, in ms
  RMSSD: the root mean square of the difference between adjacent RR intervals basically reflecting the average absolute value of the RR interval changes per stroke was sensitive to irregular heartbeats and/or heartbeat waveform marking errors, in ms

#### 2.4.2. Frequency Domain Index of HRV

  Total power (TP): it is the total variance of HRV, in ms^2^
  Very-low-frequency power (VLFP): it is the amplitude of the basic heart rate oscillation of the heart rate mode from once every 25 seconds to once every 5 minutes (0.003–0.04 Hz), in ms^2^
  Low-frequency power (LFP): it is the amplitude of the heart rate oscillation in the range of 3–9 cycles per minute (0.04–0.15 Hz), in ms^2^
  High-frequency power (HFP): it is the amplitude of the heart rate oscillation in the range of 9–24 cycles per minute (0.15–0.40 Hz, which is the typical adult breathing frequency range), in ms^2^
  LF/HF ratio: it is a ratio, usually referred to as “sympathetic-vagus” balance
  Normalization unit of low-frequency power (LFPnu): it is the ratio of LFP to HRV
  Normalization unit of high-frequency power (HFPnu): it is the ratio of HFP to HRV

### 2.5. Statistical Analyses

Each parameter was represented by the mean ± standard deviation (SD), and the data met the homogeneity of variance test and the normality test, and the paired sample *t*-test before and after itself was used. *α* = 0.05 was set, and *P* < 0.05 indicated that the difference was statistically significant using IBM-SPSS 23.0 software (SPSS Inc., Chicago, IL, USA) for statistical analyses.

## 3. Results

### 3.1. Time Domain Analysis

All the analysis results are listed in Table 1. Though WCTC16 exercise increased RR interval, SDNN, and RMSSD of SCI patients, all these changes were not statistically significant (*P* > 0.05).

### 3.2. Frequency Domain Analysis

As shown in Table 1, WCTC exercise increased TP and HFP, while decreased VLFP and LFP of SCI patients, but all these changes were not statistically significant (*P* > 0.05).

Compared with that before exercise, LFPnu and HFP peak decreased, while HFPnu increased along with LF/HF in SCI patients after WCTC16 exercise, and all these differences were statistically significant (*P* < 0.001).

## 4. Discussion

The new evidence-based exercise guidelines for SCI adults updated by the International Spinal Cord Association put more emphasis on guidelines for cardiovascular disease in SCI patients [25]. It was recommended to do aerobic exercise of medium-to-high intensity at least 2 times a week for more than 20 minutes each time and 2 times a week for each major functional muscle group, 3 groups each time medium-to-high intensity strength training (strongly recommended), to improve cardiorespiratory fitness and muscle strength. It was recommended to do aerobic exercise of medium-to-high intensity at least 3 times a week for more than 30 minutes each time (recommendation with conditions) to improve cardiovascular metabolism.

The human cardiovascular system is mainly dominated by sympathetic and parasympathetic nerves. Xiong et al. [19] found that Tai Chi could improve the HRV of middle-aged and elderly people and optimize the heart rate status. On the basis of cardiac rehabilitation, combined with Tai Chi, the sensitivity of vagus nerve reflex in patients with coronary atherosclerotic heart disease was improved, but HRV had no significant change [20]. Chen et al. [20] concluded that Tai Chi could enrich the quality of life of patients with coronary heart disease, but it had no effect on HRV.

The rMSSD is an indicator that reflects the tone of the vagus nerve [21]. In this study, the rMSSD increased slightly after WCTC16, but it was not statistically significant. As an indicator reflecting the tension of sympathetic and vagus nerve, SDNN is often used to evaluate the overall degree of autonomic nervous system damage and recovery [21]. SDNN and RR interval increased slightly following WCTC16, and they were still not statistically significant. The results of this study are consistent with the results of many authors [26–28]. The reason might be that the above indicators were not sensitive to short-term Wheelchair Tai Chi [29].

The changes of TP, VLFP, LFP, and HFP after WCTC16 exercise were not statistically significant. However, after WCTC16 exercise, the LF/HF ratio, LFPnu, and peak of HF decreased, and HFPnu increased, all of which were statistically significant. These results were consistent with the results of Buker [30].

As an objective HRV indicator, LF/HF ratio is usually used to reflect the overall sympathetic and vagal balance state [21]. In this study, the LF/HF ratio dropped from 2.24 in the rest or pre-WCTC16 period to 1.49 in the recovery or post-WCTC16 period, which was closer to the ratio of healthy adults [31]. It suggested that WCTC16 helped to restore the sympathetic-vagal balance to the level of healthy adults. Giagkoudaki et al. [32] also found that the excitability of the vagus nerve in patients with Down syndrome increased after aerobic training, the LF/HF ratio decreased (2.45∼1.72), and the balance of sympathetic-vagus nerve tension revived. Some authors showed that decreased cardiac vagus nerve activity might lead to increased mortality in patients with myocardial infarction [33]. In this study, WCTC16 increased the parasympathetic nerve activity of SCI patients. It was speculated that WCTC16 could improve autonomic nerve control and reduce mortality in SCI patients.

HFP reflects the vagus nerve regulation function [34]. In this study, HFPnu increased after WCTC16 exercise. It showed that the activity of the vagus nerve increased. LFP reflects the comprehensive regulation results of parasympathetic and sympathetic efferent nerve activity and is affected by baroreflex activity [35]. In this study, significantly reduced LFPnu suggested common regulation of vagus-sympathetic nerves and the balance function between them.

Tai Chi exercise emphasizes the training of “Qi” (bioenergy) by breathing and the mind in body. The peak of HF can reflect the influence of exercise on breathing rate [36]. The Wheelchair Tai Chi exercise also requires deep and slow breathing. In this study, the peak of HF of subjects after WCTC16 decreased from 0.29 before exercise to 0.23 during the recovery period, implying that the activity of the vagus nerve relatively increased [28, 37].

The decrease in the ratio of LFPnu and LF/HF was the result of the combined effect of decreased sympathetic nerve activity and increased parasympathetic nerve activity. It was related to the intrinsic vagus nerve reactivity, baroreceptor sensitivity, and the adaptive state accompanying spinal cord injury in patients with SCI, or a combination of the above. In addition to the abovementioned regulation of the autonomic nervous system, other factors have to be considered [38]. For example, motor stimulation interrupts the central command from the motor cortex of the brain; the stimulation of mechanoreceptors, baroreceptors, and thermoreceptors is reduced; hormone levels are disordered [39]. These are consistent with the results of related research on the effect of standing Tai Chi on heart disease in the healthy elderly [40].

WCTC16 is a moderate-to low intensity exercise for patients with complete thoracic SCI. During exercise, SCI patients have increased heart rate, blood pressure, and faster breathing, which are manifested as a relative increase in dominant sympathetic nerve activity with decreasing vagus nerve activity. After WCTC16 exercise, the parasympathetic nerve activity significantly increased, the sympathetic nerve activity decreased, and the sympathetic-vagus nerve balance improved. It showed that the body's autonomic nervous “automatic regulation” was on and a process of establishing a new balance. It is important for SCI patients with autonomic dysfunction, especially for patients whose sympathetic and parasympathetic nerve activity was reduced at the same time, to restore the dynamic balance of autonomic nervous function.

During WCTC16 exercise, it is necessary to coordinate breathing and exercise under the guidance of mind. This is consistent with the principles of cardiopulmonary rehabilitation for SCI patients. Although the primary center of the visceral reflex activity was damaged, exercise could stimulate the lower brainstem, hypothalamus, and cerebral cortex, and the combined effects developed the function of autonomic nerves and the role of system regulation. Therefore, WCTC16 had a positive role on improving the cardiopulmonary function, preventing, and treating SCI complications.

However, this study has several limitations. Firstly, the relationship between LF power of HRV and cardiac autonomic function has still been unclear. LF power seems to provide an index not of cardiac sympathetic tone but of baroreflex function [41]. This would limit the interpretation of LF and LF/HF ratio to some extent [42]. Secondly, blood pressure has some effect on HRV, but blood pressure has not been investigated in this study. Thus, it is difficult to explore the relationship between the autonomic control and blood pressure regulation [43]. So, it is incomplete to make a thorough inquiry about the mechanism of WCTC16 affecting HRV. In future, we shall design and perform correlational research studies to solve these problems.

## 5. Conclusion

The WCTC16 might enhance vagal activity and decrease sympathetic activity with balanced sympathovagal tone in patients with complete thoracic SCI. By recovering the autonomic dysfunction and hyper-reflexia causing life-threatening complication during exercise or postural changes, SCI patients could achieve a relatively “internally stable” state through complex regulation. In addition to the increase in the sympathetic nerve activity of patients during WCTC16, the parasympathetic nerve activity was significantly increased and dominant through the recovery period. In a word, WCTC16 exercise might enable the body to activate the automatic regulation of the autonomic nervous system and present a new dynamic balance.

## Figures and Tables

**Table 1:** HRV between pre- and post-WCTC16 (mean ± SD).

Parameter (unit)	Pre-WCTC16	Post-WCTC16	*t*	*P*
RR interval (ms)	604.89 ± 47.56	607.33 ± 58.12	−0.445	0.662
SDNN (ms)	34.96 ± 11.89	35.29 ± 17.76	−0.183	0.857
RMSSD (ms)	39.87 ± 19.41	41.37 ± 25.07	−0.936	0.363
TP (ms^2^)	749.00 ± 536.62	754.56 ± 586.90	−0.369	0.717
VLFP (ms^2^)	51.94 ± 21.92	50.72 ± 25.89	0.813	0.427
LFP (ms^2^)	340.06 ± 234.79	337.28 ± 258.66	0.392	0.700
HFP (ms^2^)	339.50 ± 236.88	372.28 ± 313.18	−1.455	0.164
LFPnu	61.75 ± 11.21	52.89 ± 17.48	12.753	<0.001
HFPnu	38.25 ± 11.21	46.81 ± 17.48	−10.835	<0.001
LF/HF	2.24 ± 0.68	1.49 ± 0.85	7.565	<0.001
HF peak (Hz)	0.29 ± 0.08	0.23 ± 0.08	7.526	<0.001

HRV, heart rate variability; WCTC16, 16-form Wheelchair Tai Chi; TP, total power; VLFP, power in very-low-frequency area; LFP, power in low-frequency area; HFP, power in high-frequency area; LFPnu, normalization unit of low-frequency power; HFPnu, normalization unit of high-frequency power; LF/HF, low-frequency area divided by high-frequency area; HF peak, peak of high-frequency area.

## Data Availability

The data used to support the findings of this study are available from the corresponding author upon request.

## Abbreviations

SCI: Spinal cord injury
WCTC16: 16-form Wheelchair Tai Chi
HRV: Heart rate variability
SDNN: The standard deviation of the normal RR interval
rMSSD: The root mean square of the difference between adjacent RR intervals
TP: Total power
VLFP: Very-low-frequency power
LFP: Low-frequency power
HFP: High-frequency power
LFPnu: Normalization unit of low-frequency power
HFPnu: Normalization unit of high-frequency power.
